# Perinatal Distress During COVID-19: Thematic Analysis of an Online Parenting Forum

**DOI:** 10.2196/22002

**Published:** 2020-09-07

**Authors:** Bonnie R Chivers, Rhonda M Garad, Jacqueline A Boyle, Helen Skouteris, Helena J Teede, Cheryce L Harrison

**Affiliations:** 1 Monash Centre for Health Research and Implementation School of Public Health and Preventive Medicine Monash University Clayton Australia; 2 Department of Obstetrics and Gynaecology Monash Health Clayton Australia; 3 Diabetes and Vascular Medicine Unit Monash Health Clayton Australia

**Keywords:** pregnancy, perinatal, maternal, COVID-19, communication, social support, qualitative research, mental health, health information, online support, thematic analysis, sentiment analysis, word frequency

## Abstract

**Background:**

The COVID-19 global pandemic has impacted the whole of society, requiring rapid implementation of individual-, population-, and system-level public health responses to contain and reduce the spread of infection. Women in the perinatal period (pregnant, birthing, and postpartum) have unique and timely needs for directives on health, safety, and risk aversion during periods of isolation and physical distancing for themselves, their child or children, and other family members. In addition, they are a vulnerable group at increased risk of psychological distress that may be exacerbated in the context of social support deprivation and a high-risk external environment.

**Objective:**

The aim of this study is to examine the public discourse of a perinatal cohort to understand unmet health information and support needs, and the impacts on mothering identity and social dynamics in the context of COVID-19.

**Methods:**

A leading Australian online support forum for women pre- through to postbirth was used to interrogate all posts related to COVID-19 from January 27 to May 12, 2020, inclusive. Key search terms included “COVID,” “corona,” and “pandemic.” A three-phase analysis was conducted, including thematic analysis, sentiment analysis, and word frequency calculations.

**Results:**

The search yielded 960 posts, of which 831 were included in our analysis. The qualitative thematic analysis demonstrated reasonable understanding, interpretation, and application of relevant restrictions in place, with five emerging themes identified. These were (1) heightened distress related to a high-risk external environment; (2) despair and anticipatory grief due to deprivation of social and family support, and bonding rituals; (3) altered family and support relationships; (4) guilt-tampered happiness; and (5) family future postponed. Sentiment analysis revealed that the content was predominantly negative (very negative: n=537 and moderately negative: n=443 compared to very positive: n=236 and moderately positive: n=340). Negative words were frequently used in the 831 posts with associated derivatives including “worried” (n=165, 19.9%), “risk” (n=143, 17.2%), “anxiety” (n=98, 11.8%), “concerns” (n=74, 8.8%), and “stress” (n=69, 8.3%).

**Conclusions:**

Women in the perinatal period are uniquely impacted by the current pandemic. General information on COVID-19 safe behaviors did not meet the particular needs of this cohort. The lack of nuanced and timely information may exacerbate the risk of psychological and psychosocial distress in this vulnerable, high-risk group. State and federal public health departments need to provide a central repository of information that is targeted, consistent, accessible, timely, and reassuring. Compensatory social and emotional support should be considered, using alternative measures to mitigate the risk of mental health disorders in this cohort.

## Introduction

COVID-19, a novel strain of coronavirus, is an acute, highly infectious virus that has affected tens of millions of people and has caused close to 1 million deaths as of August 2020 [1]. The disease is spread directly through respiratory droplets from the mouth or nose. It is estimated that up to 40% of transmission is presymptomatic, with an average incubation period of 5-6 days [2]. The vast majority of those who are infected with COVID-19 experience mild to moderate illness arising from a cluster of mild symptoms including fever, dry cough, and lethargy [3]. Currently, there is no effective COVID-19 medical prophylaxis and limited treatment, mandating a rigorous individual-, population-, and system-level public health policy and behavioral change response to minimize transmission [4].

COVID-19 originated in Wuhan, China, with the first cases reported in December 2019 [5], and was officially declared as a public health emergency of international concern on January 30, 2020, by the World Health Organization. The first Australian confirmed case of COVID-19 was on January 25, 2020, in Victoria, originating from Wuhan, China, subsequently resulting in the closing of Australian borders to all nonresidents in March [6]. Physical distancing rules were imposed on March 21, 2020, with the associated closure of all *nonessential* services including retail outlets, cafés, restaurants, schools, recreational facilities, and playgrounds [7]. To assist in physical distancing, additional measures including working, studying, or completing school from home were imposed; social gatherings were banned, and stringent restrictions on individual movement were put in place [7]. These public health policies profoundly impacted individual- and population-level health, disrupted normal social interactions, and contributed to economic insecurity.

As COVID-19 continues to disrupt human interactions, published data on the risk, transmission, and health outcomes of specific populations, including women in the perinatal period and their neonate, are evolving, yet are currently inconclusive [8-10]. Women within the perinatal period are a vulnerable population, both physiologically, with changes during pregnancy that reduce immunity [11], and psychologically, with increased risk of psychological distress including stress, anxiety, and depression [12-15], all of which may increase maternal and neonatal morbidity [16]. Consequently, it is recognized that women during this period require unique and specific health, support, and information needs to avoid stress [17]. Currently, the impact of COVID-19 on such needs and associated levels of distress is poorly understood, yet is critical in ascertaining adverse implications, as well as in identifying strategies to protect and optimize women’s psychosocial health during this time. Therefore, this study aims to understand the sentiment and impacts to emotional well-being as well as the unmet information and support needs arising from changes to social dynamics and support in a perinatal cohort during the COVID-19 pandemic.

## Methods

### Overview

We conducted an observational, qualitative analysis of online discussions within a leading Australian forum for new or expecting parents. The most popular Australian pre- and postbirth forum was identified by searching the term “new mum forum” in Google. The top 10 (first page) results were assessed, and all websites with publicly available forums (n=7) were analyzed using a website analytics tool (Alexa, Amazon.com). This software was used to determine the global page views, global rank, and Australian rank of the 7 websites with publicly available forums. The highest ranked website for Australian users was identified and used as the sampling platform for this study. To confirm this website’s suitability for this study, member requirements were assessed to ensure forum users were new or expecting mothers.

Within the selected forum, the search function was used to identify user-generated content relating to COVID-19. No date restrictions were applied in the search with posts collected on May 12, 2020. We searched for website content using key search terms including “COVID,” “corona,” or “pandemic.” The search identified articles, comments, and posts, which was then narrowed to posts. All posts were extracted in a deidentified format into a Word (Microsoft Corporation) document, which included the post title, date, and content. The inclusion criteria were posts related to COVID-19 up until May 12, 2020 (inclusive). The exclusion criteria were posts that included a title only (content had been deleted), that did not relate to COVID-19, and that were duplicate posts (original post was collected once).

### Analysis

Data was processed using NVivo Pro 12 (QSR International) software [18]. Analysis comprised three phases, including thematic analysis, sentiment analysis, and word frequency calculations of stemmed words. Thematic analysis was undertaken using a modified grounded theory approach that was informed by Braun and Clarke’s [19] six phase approach. A single researcher (BC) became familiar with the data, generated initial codes, and searched for themes. The team then collaborated to discuss themes and a >25% check of themes was conducted by two additional researchers (CH and RG). To support the themes identified, NVivo Pro 12 automatic sentiment analysis and a text frequency search were run to identify emotional indicators. NVivo searched for expressions of sentiment in the source material then used predefined scores for words classified as containing sentiment [20]. Words are considered in isolation, and the program then determines the sentiment of the paragraph as a calculation of each word containing the sentiment. Sentiment results include the number of references (paragraphs with sentiment) that are categorized as very positive (VP), moderately positive (MP), moderately negative (MN), and very negative (VN). A single researcher (BC) conducted a >10% cross-check of the sentiment results. Word frequency calculations were used to identify all stemmed words (minimum 3 letters) used 50 times or more. These words were screened by a single researcher (BC) to identify negative words. The frequency of key terms used was divided by the number of total posts to derive an overall percentage, and a weighted percentage on the total word count was calculated by NVivo Pro 12.

### Ethics

Ethics approval for this study was granted by the Monash Health (RES-19-0000-291A) and Monash University (Project no 20196) Human Research Ethics Committees. Although ethical oversight of publicly available data is not strictly required, the authors sought approval as per Monash University protocol.

## Results

### Overview

A total of 960 posts were identified using the search terms (“corona” n=589, “COVID” n=257, and “pandemic” n=114). As per the exclusion criteria, 114 posts were excluded, resulting in a final sample of 831 unique posts. The first relevant post identified was dated January 27, 2020.

### Thematic Analysis

We identified five themes from the analyzed content: (1) heightened distress related to a high-risk external environment; (2) despair and anticipatory grief due to deprivation of social and family support, and bonding rituals; (3) altered family and support relationships; (4) guilt-tampered happiness; and (5) family future postponed.

#### Theme 1: Heighted Distress Related to a High-Risk External Environment

Women expressed concerns and unease due to a range of factors such as the lack of access to particular information on risk (eg, risk during pregnancy, risk to baby in utero, risk to a new born baby, risk from the hospital environment, risk to mental health from reduced social supports). They asked questions within the discussion forum, such as should pregnant women cease working, what is the risk of COVID-19 to unborn or newly born babies, should working partners isolate from their pregnant spouse or from new babies, are pregnant women at increased risk, or should I be leaving the house? Many women were unable to locate information to fully answer these concerns and sought confirmation of both their concerns and their risk reduction actions from their peers within the forum.

Women who stated they were close to giving birth demonstrated significant levels of worry in relation to the safety of antenatal appointments and the hospital environment. Some indicated they were considering a home birth, and others stated they were considering not attending antenatal visits due to fear of contracting COVID-19.

I am due to get my NT scan done...I am getting nervous about having to travel there from a small rural town to have it done. I am considering whether I should get it done or not worry.

One woman stated that, although she did not want to be “alarmist” the lack of information on hospital safety made her feel “vulnerable.” Others stated that “being pregnant during this time is so scary...it's certainly not how I envisioned it to be going or to end,” and another woman said, “I almost don’t want to give birth right now.”

Women said they found it difficult to disengage with the constant worry and concern about COVID-19 with one woman stating that

...all I’m thinking and dreaming about is COVID-19! I’m super paranoid, I feel like even getting groceries, I have anxiety.

Many women said they had disengaged with mainstream news sources as they found these to be fear inducing. Posts reflected heightened levels of worry and stress in this cohort. One woman stated, “I feel the most anxious, overwhelmed, isolated, out of control as I've ever felt before.”

#### Theme 2: Despair and Anticipatory Grief due to Deprivation of Social and Family Support, and Bonding Rituals

Due to the unforeseen restrictions from the pandemic, usual perinatal social rituals such as baby showers, celebrations, or gender reveals were not possible. In addition, areas where women heavily rely on social support, such as multiple support persons during birth and postbirth, and family or social support providing physical, psychological, and social care, were denied to this group of women. When one woman was told she was not allowed to have her partner with her during a routine scan she said, “I am really sad as it’s our first pregnancy and these scans are small ways where our husbands can take part in our pregnancy.” Many expressed a range of feelings such as sadness, anger, and a sense of loss. One woman stated “the happiest time in our lives is being over shadowed by this virus.” Another said “I’ve played many times over, my siblings and parents coming to hospital to meet my first child and that was a once in a life time moment for me; something I think very special that I won’t get anymore.” Upon reading about the COVID-19–related grief, one woman said:

totally made me realise that I'm grieving the loss of a normal pregnancy...I checked out the five stages of grief, and I'm definitely going through the emotions they list! Anger and bargaining - hopefully I'll move on to true acceptance soon.

#### Theme 3: Altered Family and Support Relationships

Customary ways of strengthening relationships with family and social networks during pregnancy and early motherhood were denied to this group of women due to the demands of isolation. Many of the women discussed conflicts with family members over interpretations of physical distancing rules. This conflict was expressed by one woman as:

I don’t understand how they whinge that they can’t see their grandson yet continue to do all the things THAT ARE THE REASON we don’t want our son around them...Has anyone else had to deal with family not taking this virus seriously?

The impact of COVID-19 isolating new mothers from their support and family networks, and the unique need to protect the health of grandparents reduced social support for new mothers and resulted, in some cases, in unanticipated interfamily conflict. This had the effect of dividing some families rather than the bonding experiences that many may have anticipated.

#### Theme 4: Guilt-Tampered Happiness

Many women expressed feelings of guilt due to the contrast between the positivity and happiness they were experiencing at the news of their pregnancy or arrival of their child, and the difficult situation of many others in the community.

I feel a bit guilty about wanting to share my good news. I...have been looking forward to sharing about our baby especially after I was told I would not be able to have kids at all.

Other women identified guilt relating to their wishes to experience baby showers or events that their peers have formerly enjoyed:

this might sound a little selfish but it’s our first and I was looking forward to the gifts and games etc. I hope by August maybe we can have a small gathering.

Although another woman stated she was feeling guilty for thinking about her pregnancy plans. “I hope everyone is coping with the current covid crisis - I feel a bit guilty thinking about IVF.”

#### Theme 5: Family Future Postponed

Many women discussed their concerns about family planning and of wanting to postpone plans to extend their family. “I’ve heard so much information (and misinformation) the last few weeks it’s really made me fearful about trying to conceive with all the madness around.” Another posed the questions:

[is anyone] temporarily pausing trying to conceive whilst our world is being rattled by corona? Has it changed your timeline in terms of when you want baby to born? Have you decided to wait or maybe not even have anymore?

### Sentiment and Word Frequency Analysis

There were 1556 references of sentiment. Of these, most were found to be within the negative range, with 980 (63.0%) references classified as negative (VN: n=537 and MN: n=443) compared with 576 positive references (VP: n=236 and MP: n=340). A cross-check of sentiment results found that less than 10% of sentiment references were coded to incorrect categories.

A list of words used 50 times or more (n=189) was examined to screen for negative words. Multiple negative emotive stem words (n=7) were identified (Table 1). Percentages derived from the total number of posts provide an indication of the spread of these words within the data set.

**Table 1:** Word frequency in total posts (N=831).^a^

Word	Similar word	Frequency, n (%)	Weighted percentage (%)
Worrying	Worried, worries, worry	165 (19.9)	0.32
Risk	Risking, risks	143 (17.0)	0.27
Anxiety	Anxieties, anxious, anxiousness	98 (11.7)	0.19
Concerns	Concern, concerned, concerning	74 (8.8)	0.14
Stress	Stressed, stressful, stressing	69 (8.2)	0.13
Struggling	Struggle, struggled	59 (7.0)	0.11
Scare	Scares, scared, scaring	55 (6.6)	0.11

^a^Percentage calculations were derived based on the word count in context of the total posts (identifying an approximate number of posts including these words; however, this cannot account for words used multiple times in one post) and as a weighted percentage of the total word count.

## Discussion

### Principal Results

The rapidly evolving COVID-19 pandemic is a global emergency, requiring unprecedented public health policy changes and behavioral adherence to limit viral spread. This vital public health response comes at significant health, societal, and economic cost, and is anticipated to have a profound effect on emotional well-being and mental health. During the perinatal period, women have specific health care and emotional needs, and are vulnerable to mental health challenges. Understanding the unique perinatal information needs and impact of losing support networks is, therefore, critical in informing public health and health care system’s interventions for the well-being of women and their families.

Our results demonstrate a significant response to the pandemic within this cohort, with an average of 275 posts per month directly related to COVID-19 across the evaluation period. Thematic analysis captured five themes: heightened distress related to a high-risk external environment; despair and anticipatory grief due to deprivation of social and family support, and bonding rituals; altered family and support relationships; guilt-tampered happiness; and a family future postponed. Sentiment analysis showed heightened negativity with high frequency use of negative terms including “stress,” “anxious,” “worry,” and “risk,” with emergent qualitative themes identified related to anxiety, grief, guilt, social support, and disrupted family planning.

To enable rapid evaluation and dissemination of findings, we chose to examine a leading online Australian perinatal forum, accessed broadly by reproductive-aged women across preconception, pregnancy, postpartum, and into early parenting and childhood. Online support groups have previously been shown to be commonly accessed by women during this period. They provide an accessible, peer-to-peer opportunity for information sharing, individualized information seeking, and social and emotional support from women in a comparable life stage [21,22]. Accessibility and anonymity provided by online forums facilitates a disinhibited expression of feelings without concern of consequences or conflict from close connections. Engagement with and preference for interacting with peers in an online forum may reduce engagement with other forms of media. Indeed, this is supported by our thematic analysis showing many women reporting disengagement with mainstream news sources, as they found these to be fear-provoking.

Anticipatory grief for loss of social and family affirming opportunities during the perinatal period was emphasized as a consequence of physical distancing measures in this cohort. Pregnancy and birth are often a celebratory period for women and are associated with joy experienced by both the parents and close family members. Traditional rituals and milestones including the early pregnancy growth and development scans, baby shower celebrations, and hospital visits by family and grandparents were all effectively denied in accordance with physical distancing and personal safety measures. Such milestones present significant opportunities for social support and likely play a role in strengthening family and partner relationships, in turn increasing well-being, self-efficacy, and coping during this time. Disruption or removal of these milestones is, therefore, likely to impact well-being, potentially increasing feelings of isolation and consequently driving conflict due to anger and frustration, exacerbating pre-existing negativity within the wider external environment.

Conversely, many women also expressed guilt as a result of feeling joy and happiness related to their pregnancy or impending birth. This *paradox of guilt* in the context of COVID-19 is reportedly similar to the phenomenon of survivor’s guilt, an experience of immense guilt toward surviving a traumatic event that others in a given population did not [23]. Here, women felt happiness yet felt exclaiming such feelings were inappropriate in the context of widespread illness, grief, and loss at a population level. Although this may not be directly termed as survivor’s guilt, a correlation is plausible. Women may also be disproportionately affected by feelings of guilt in this context, with research showing increased concern for the health of others including older family members and children, rather than the health and well-being of themselves [24].

Our results show an interruption in family planning as a consequence of COVID-19. This may be due to several factors including economic and employment insecurity or fear of direct health impacts. The sudden closure of many sectors has profoundly impacted the economy, contributing to the largest rise in unemployment rate since the depression, with women disproportionately impacted [25]. Hesitancy related to health outcomes during pregnancy may also impact family planning. Although women are generally more resistant to viral infections than men, physiological changes that occur during pregnancy increase vulnerability to severe infections due to a reduced immune response. A recent systemic review of the first 108 pregnant women infected with COVID-19 found a majority of case reports occurred in the last trimester with associated severe maternal morbidity [8]. This included a cesarean section rate of 91% across cases, predominantly due to fetal distress, with 1 intrauterine and 1 neonatal death recorded. Despite the majority of women recovering without major complication, the authors concluded that mother-to-baby transmission of COVID-19 remains unclear [8], with similar reports published elsewhere [9,10].

Our sentiment analysis revealed increased negativity during the evaluation period, reflected by ~60% of posts related to distress including anxiety, worry, and risk. These results are similar to recent research evaluating user sentiment on Twitter during the COVID-19 pandemic [26]. Following a brief analysis of daily tweets related to four key emotions, the authors concluded fear was most strongly represented, followed by anger; joy; and, lastly, sadness. Increased negativity on social media is likely to be indicative, in part, of the broader population’s overall sentiment. Indeed, this is supported by recent population-based research by the UK Office of National Statistics, reporting a doubling in high levels of anxiety between October 2019 to April 2020, compared with an earlier reference period [27]. Notably, women appear disproportionately affected, with 24% higher frequency of anxiety than men overall. Authors postulated that increased anxiety in women may be related to financial impacts, including a reduced likelihood of employment due to child rearing and gender inequities in salary. Increased anxiety and depressive symptoms in women are also common during pregnancy [15], and recent preliminary research reported a two-fold increase during COVID-19 [28]. The impact of social isolation was reported as a significant predictor of increased depression on a regression analysis [28]. Pregnancy, birth, and the postpartum periods are life stages requiring increased levels of social support, and reduced support at this time has been shown to have detrimental impacts on maternal mental health outcomes [29]. Women in this cohort frequently reported having reduced access to social support networks such as partners (ie, essential workers or those requiring quarantine due to business-related travel), family members, significant others, social networks, mothering groups, and health care professionals. In turn, this may have increased engagement with the forum as a source of like-minded support and comfort.

The women using the discussion forum demonstrated reasonable understanding and knowledge of relevant public health restrictions. This is encouraging with respect to behavior changes in response to the COVID-19 pandemic, as it indicates successful public health communication to the general community. However, women in the perinatal period are highly motivated to seek nuanced information that reduces risk to the mother, unborn child, or new baby. This includes information relating to self-isolation and safety while pregnant, employment implications if unable to work from home, the risks of COVID-19 infection at health facilities during perinatal medical visits, restrictions on birthing supports, and isolation from working partners. Women indicated information was sought from multiple sources but indicated, however, that it did not meet their current needs and was either general in nature, ambiguous, or inconsistent. It is critical that the information needs of this cohort are understood and met to reduce the risk of mental health disorders in an already high-risk group and to reduce the risk of avoidance of health care monitoring. This is particularly imperative during pregnancy with some women indicating they would postpone or avoid antenatal visits, which may lead to negative health outcomes for both mother and baby. State and federal public health departments need to provide a central repository of information for this cohort that is accessible, timely, and reassuring. It is also important to understand that women are using discussion forums as their primary source of information in lieu of official sources.

### Limitations

The following limitations should be considered when interpreting these findings. Anonymized data was interrogated and, therefore, demographical and geographical information about the user could not be obtained that may influence the user’s engagement with the forum, as well as their perception and reaction to the COVID-19 pandemic. However, in accessing real-world nonidentified data, information is less likely to be influenced by social desirability or recall bias. We cannot confirm all posts were written by women in the perinatal period, however, forum users must enter an expected due date, child’s birth date, or declare that they are trying to conceive to become a member of the forum.

Additionally, the real-world implications of users’ online posts are unclear, and detrimental effects may be overemphasized in the absence of sufficient data pertaining to real-world behavior and state of mind. Due to early inconsistency in pandemic-related terminology, users may have used other keywords to describe COVID-19 that were not collected in this study. Finally, due to site management and restrictions relating to the seeking or provision of medical advice via the forum, posts may have been deleted before data was collected. In addition, the limitations of the software used in the sentiment analysis must be acknowledged, such as the inability to recognize sarcasm, double negatives, slang, dialect variations, idioms, and ambiguity. However, to address this limitation, we cross-checked the results and determined less than 10% were incorrectly classified. We believe this to be one of the first evaluations of the social impacts of COVID-19 in mothers or pregnant women in Australia [24,30-33]. This paper provides critical insights into the unanticipated impacts of this pandemic on a high need’s perinatal cohort.

### Conclusion

The perinatal period involves a major life transition requiring increased levels of social, emotional, and health professional support. Our results demonstrate pregnant women and new mothers are uniquely impacted by the COVID-19 pandemic. General information on COVID-19 safe behaviors does not appear to meet the needs of this population. The lack of nuanced and timely information appears to have exacerbated the risk of psychological and psychosocial distress in this vulnerable group who demonstrate heightened distress, reduced social and emotional support, anticipatory grief, increasing interfamily conflicts, and direct impacts on family planning behaviors. These findings suggest the need for targeted, consistent, accessible, and timely information on risk and risk aversion strategies, and adoption of strategies to de-escalate anxiety and concern. It also suggests that support strategies are needed to compensate for the loss of family, social support, and health professional contact, and lastly, that mental health interventions tailored to the unique needs of this cohort are likely important during the pandemic and for related public health policies.

## Abbreviations

MN: moderately negative
MP: moderately positive
VN: very negative
VP: very positive
